# The human Vδ2^+^ T-cell compartment comprises distinct innate-like Vγ9^+^ and adaptive Vγ9^-^ subsets

**DOI:** 10.1038/s41467-018-04076-0

**Published:** 2018-05-02

**Authors:** Martin S. Davey, Carrie R. Willcox, Stuart Hunter, Sofya A. Kasatskaya, Ester B. M. Remmerswaal, Mahboob Salim, Fiyaz Mohammed, Frederike J. Bemelman, Dmitriy M. Chudakov, Ye H. Oo, Benjamin E. Willcox

**Affiliations:** 1 Cancer Immunology and Immunotherapy Centre, Institute of Immunology and Immunotherapy, University of Birmingham, Birmingham, B15 2TT UK; 2 Centre for Liver Research and NIHR Biomedical Research Unit in Liver Disease, Institute of Immunology and Immunotherapy, University of Birmingham, Birmingham, B15 2TT UK; 3 Shemyakin-Ovchinnikov Institute of Bioorganic Chemistry, Russian Academy of Science, Moscow, 117997 Russia; 4 Centre for Data-Intensive Biomedicine and Biotechnology, Skolkovo Institute of Science and Technology, Moscow, 143026 Russia; 5 Department of Experimental Immunology, Academic Medical Center, Amsterdam, 1105 AZ The Netherlands; 6 Renal Transplant Unit, Division of Internal Medicine, Academic Medical Center, Amsterdam, 1105 AZ The Netherlands; 7 Central European Institute of Technology, Masaryk University, Brno, 625 00 Czech Republic; 8 Pirogov Russian National Research Medical University, Moscow, 117997 Russia

## Abstract

Vδ2^+^ T cells form the predominant human γδ T-cell population in peripheral blood and mediate T-cell receptor (TCR)-dependent anti-microbial and anti-tumour immunity. Here we show that the Vδ2^+^ compartment comprises both innate-like and adaptive subsets. Vγ9^+^ Vδ2^+^ T cells display semi-invariant TCR repertoires, featuring public Vγ9 TCR sequences equivalent in cord and adult blood. By contrast, we also identify a separate, Vγ9^−^ Vδ2^+^ T-cell subset that typically has a CD27^hi^CCR7^+^CD28^+^IL-7Rα^+^ naive-like phenotype and a diverse TCR repertoire, however in response to viral infection, undergoes clonal expansion and differentiation to a CD27^lo^CD45RA^+^CX_3_CR1^+^granzymeA/B^+^ effector phenotype. Consistent with a function in solid tissue immunosurveillance, we detect human intrahepatic Vγ9^−^ Vδ2^+^ T cells featuring dominant clonal expansions and an effector phenotype. These findings redefine human γδ T-cell subsets by delineating the Vδ2^+^ T-cell compartment into innate-like (Vγ9^+^) and adaptive (Vγ9^−^) subsets, which have distinct functions in microbial immunosurveillance.

## Abstract (web summary)

Human Vδ2^+^ γδ T cells are thought to be an innate-like T-cell population. Here the authors show the Vδ2^+^ compartment contains both innate-like Vγ9^+^ and an adaptive Vγ9^-^ subset that undergoes clonal expansion during viral infection and can infiltrate liver tissue.

## Introduction

γδ T cells have coevolved alongside B cells and αβ T cells in the vertebrate immune system for almost 450 million years^1^. They provide anti-microbial^2^ and anti-tumour immunity^3^, but whether they occupy an innate-like or adaptive immunological niche has remained unclear. Notably, αβ T cells incorporate a group of unconventional T cells, including mucosal-associated invariant T (MAIT) cells and invariant natural killer T (iNKT) cells that recognise antigens in the context of single MHC-like proteins (MR1 and CD1d), and display a semi-invariant T-cell receptor (TCR) repertoire, suggestive of an innate-like biology whereby TCR sensitivity is retained but the γδ TCR may arguably function as a surrogate pattern recognition receptor^4^. Notably, studies in mice have suggested that innate-like γδ T-cell development in the thymus can occur via distinct pathways involving agonistic signals^5^. In addition, recently, Wencker et al.^6^ have suggested that following TCR triggering during development, mouse innate-like T cells may transition to a state of TCR hyporesponsiveness in which they preferentially respond to TCR-extrinsic stimuli such as cytokine exposure.

Human γδ T cells are often delineated into Vδ2^+^ and Vδ2^−^ subsets^7^. Vδ2^−^ γδ T cells have been directly implicated in anti-viral and anti-tumour immunity^3,8^ and utilise germline-encoded antigen receptors also present on innate-like lymphocytes, including NKG2D and NKp30^9,10^. However, recent evidence has suggested that they may adopt a TCR-dependent adaptive immunobiology, based on highly clonotypically focused expansions alongside differentiation from a naive to effector phenotype^11^ and perturbations in clonal expansion upon cytomegalovirus (CMV) infection in post-stem cell transplant patients^12^. Conversely, Vδ2^+^ T cells are arguably the prototypic unconventional T cell, typically co-expressing Vγ9 TCR chains and representing the major γδ subset in adult peripheral blood^13^. Vγ9^+^ Vδ2^+^ T cells respond to prenyl pyrophosphate metabolites (phosphoantigens, or P-Ags) produced either by the host mevalonate pathway (isopentenyl pyrophosphate, IPP) or microbial non-mevalonate pathway ((*E*)-4-Hydroxy-3-methyl-but-2-enyl pyrophosphate, HMB-PP)^14^, which are sensed in the context of butyrophilin 3A1 (BTN3A1)^15–17^. They mount important anti-microbial immune responses, including against *Mycobacterium tuberculosis*^13^ and *Plasmodium falciparum*^18^, and drive αβ T-cell responses^19,20^.

Previously, the extrathymic expansion of Vγ9^+^ Vδ2^+^ T cells observed in adult peripheral blood, which is proposed to result from exposure to P-Ag-producing microbes encountered after birth^21,22^ and thought to involve clonotypic expansion^23,24^, arguably suggests an adaptive immunobiology. However, Vγ9^+^ Vδ2^+^ T cells are highly enriched in foetal peripheral blood and display restricted complementarity-determining region (CDR) 3 γ9 usage early in gestation^25^, more consistent with an innate-like pre-natal repertoire of Vγ9^+^ Vδ2^+^ T cells.

To better understand human Vδ2^+^ γδ T-cell immunobiology we carried out a dedicated analysis of their clonotypic diversity and cellular phenotype. Our findings suggest Vδ2^+^ T cells can be delineated into two discrete subsets: Vγ9^+^ Vδ2^+^ T cells adopt a predominantly innate-like biology originating in neonatal development and allowing a degree of clonotypic plasticity, whereas Vγ9^−^ Vδ2^+^ T cells adopt a distinct adaptive immunobiology, including focused clonal expansions and differentiation evident both in peripheral blood and solid tissues, and generated in response to acute viral infection. These findings revise our understanding of human γδ T cells, and prompt further investigation of the adaptive immunity provided by the Vγ9^−^ Vδ2^+^ subset.

## Results

### Adult and neonatal Vδ2^+^ T cells have similar TCR diversity

**Fig. 1:**
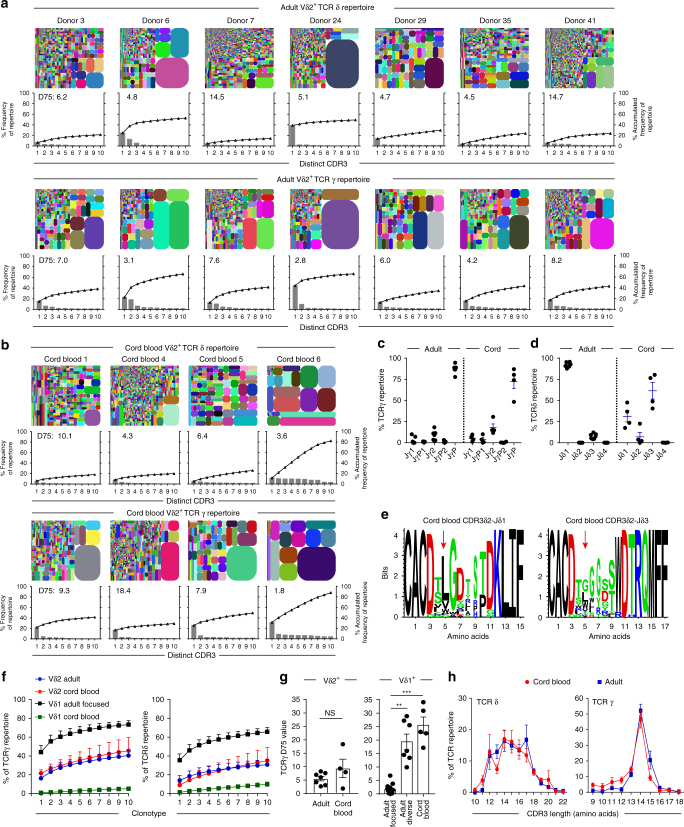
Adult and cord blood Vδ2^+^ TCR repertoires are broadly similar. **a** Tree maps show each adult donor’s Vδ2^+^ TCR repertoire, with each CDR3 clonotype as a coloured segment (each coloured CDR3 segment is chosen randomly and does not match between plots) plotted in relation to the total repertoire size and accompanying clonotype frequency graphs showing the individual clone frequency (left *y* axis) and the accumulated frequency for the 10 most prevalent clonotypes (right *y* axis). Inset into each graph are D75 repertoire diversity metrics (measuring the percentage of clonotypes required to occupy 75% of the total TCR repertoire). **b** Tree maps showing TCR γ and δ CDR3 clonotypes, accumulated frequency graphs and D75 metric from cord blood Vδ2^+^ T cells. **c** Jγ and **d** Jδ segment usage in Vδ2^+^ TCR repertoires from adult peripheral blood (*n* = 7) and cord blood samples (*n* = 4). **e** Logo analysis of amino acid enrichment at each position in neonatal Vδ2–Jδ1 CDR3δ (left) and Vδ2–Jδ3 CDR3δ (right) sequences. Analysis was confined to the 10 most abundant CDR3δ2 sequences of 13–16 amino acid length. The different amino acids are coloured according to physicochemical properties (acidic (red); basic (blue); hydrophobic (black); and neutral (green)). Red arrows indicate position 5 in the CDR3 sequence (see Methods section). **f** Comparison of accumulated frequency curves generated from the 10 most prevalent TCRγ (left) and δ (right) clonotypes in Vδ2^+^ and Vδ1^+^ TCR repertoires (Vδ1 cohort data analysed from^11^) from adult peripheral blood (Vδ2^+^, *n* = 7 and Vδ1^+^, *n* = 13) and cord blood (Vδ2^+^, *n* = 4 and Vδ1^+^, *n* = 5). **g** Comparison of TCRγ D75 metrics from adult peripheral blood and cord blood Vδ2^+^ (adult: *n* = 7; cord blood: *n* = 4) and Vδ1^+^ repertoires (adult focused: *n* = 13; adult diverse: *n* = 7; cord blood: *n* = 5). **h** Comparison of the CDR3 length profiles in Vδ2^+^ TCRδ and γ repertoires from adult peripheral blood (*n* = 7) and cord blood (*n* = 4). Error bars indicate means ± SEM; **P < 0.01; ***P < 0.001; *p*-values were determined by Student's *t*-test (**g**: left) and Kruskal–Wallis test (ANOVA) with Tukey’s post hoc testing (**g**: right). NS not significant

Consistent with previous studies, we confirmed that human cord blood Vδ2^+^ γδ T cells were present at relatively low levels, but were increased as a proportion of peripheral T cells by adulthood^22,26^ (Supplementary Fig. 1a), resulting in an adult human Vδ2^+^ T-cell repertoire that is uniformly responsive to P-Ag metabolites (Supplementary Fig. 1b). To address whether Vδ2^+^ T-cell expansion from the neonatal pool is highly clonally focused, as for Vδ1^+^ T cells^11^, we performed a TCR repertoire analysis restricted to the Vδ2^+^ T-cell population (Fig. 1a, b). Indicative of successful Vδ2^+^ γδ T-cell sorting, TCRδ repertoires entirely comprised Vδ2 chain usage (Supplementary Fig. 1c). Consistent with previous findings, TCRγ repertoires were predominantly composed of Vγ9 chains (Supplementary Fig. 1d) and the joining region JγP (Fig. 1c), and neonatal Vδ2 chains preferentially used joining region Jδ3^27^, whereas Jδ1 was more commonly utilised in adults^28^ (Fig. 1d). TCRδ-mediated responses to P-Ag have been associated with hydrophobic amino acid residues (LVW) at position 5 in CDR3δ2 sequences^29^, and this is observed in adult Vδ2 CDR3 repertoires^11^. We found that although neonatal Vδ2–Jδ3 sequences generally lacked this motif, and overall were enriched relative to Vδ2–Jδ1 sequences for neutral residues at position 5, particularly Gly, some Vδ2–Jδ3 sequences contained the hydrophobic residue Leu, whereas Vδ2–Jδ1 sequences, although rarer in neonates, were highly enriched for hydrophobic residues at position 5 (Fig. 1e).

We next analysed Vδ2^+^ T-cell repertoires using approaches we previously applied to the Vδ1^+^ compartment^11^. Tree plot analysis revealed the presence of some relatively prominent clonotypes in adult Vδ2 TCRγ (between 12 and 47%) and TCRδ (between 1.8 and 39%) repertoires (Fig. 1a). The ten most prevalent TCRγ clonotypes in each donor formed a substantially smaller portion (*P* = 0.003; Mann–Whitney) of the Vδ2^+^ T-cell repertoire (mean 40.5% of total Vδ2 reads) than expanded Vδ1 clonotypes (mean 73.42% of total Vδ1 reads^11^). Unlike the Vδ1 compartment, similarly prominent clonotypes were also present in cord blood Vδ2^+^ TCRγ (between 18 and 34%) repertoires (Fig. 1a, b). Analysis of the cumulative frequency curves of the 10 most prevalent CDR3 sequences highlighted both adult and cord blood Vδ2^+^ TCR repertoires exhibit an intermediate degree of clonotypic focusing, relative to cord blood Vδ1 (low focusing) and clonally expanded adult Vδ1 repertoires (high focusing; Fig. 1f). Analysis of D75 diversity metrics (the percentage of clonotypes required to occupy 75% of TCR repertoire) highlighted that in Vδ2^+^ T-cell repertoires diversity did not generally differ between adult and cord blood Vδ2^+^ repertoires (Fig. 1g and Supplementary Fig. 1e). Moreover, Vδ2^+^ TCR repertoire D75 metrics were also independent of the level of diversity seen in matched Vδ1^+^ TCR repertoires (Supplementary Fig. 1f). Finally, and consistent with a broadly similar Vδ2^+^ repertoire in cord and adult blood, the CDR3 length profile of TCRδ and TCRγ repertoires were highly comparable between cord blood and adult (Fig. 1h). Therefore, while some variability in the level of focusing is observed in both settings, the neonatal and adult Vδ2^+^ repertoires are broadly similar in diversity, in terms of both accumulated clonotype frequency, D75, and CDR3 length analysis.

### Public Vγ9^+^ CDR3s span adult and neonatal Vδ2^+^ T cells

**Fig. 2:**
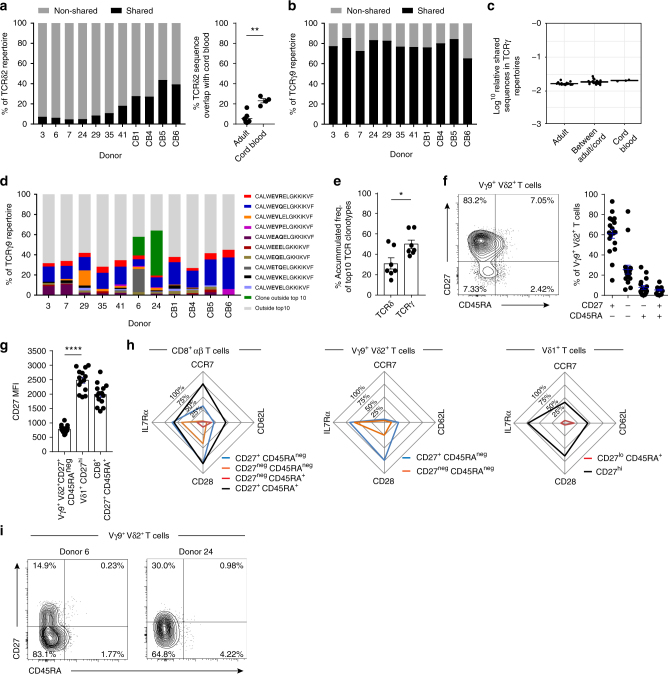
Vδ2^+^ TCR repertoires are formed of public clonotypes. **a** Percentage of CDR3δ sequences (amino acid) shared between > 2 donors within both adults and cord blood (left) and sequences shared with cord blood only (right). **b** Percentage of CDR3γ9 sequences (amino acid) shared between >2 donors (public sequences). **c** Comparison of the sequence overlap (relative publicity) of TCRγ9 repertoires in adult peripheral blood donors (*n* = 7) or cord blood samples (*n* = 3) and then between both groups. **d** Frequency of each of the 10 most common clonotypes in each donor’s TCRγ9 repertoire, with the addition of exceptional expanded shared clonotypes usually found outside the top 10 (dark green). **e** Comparison of the accumulated repertoire frequency occupied by the first 10 clonotypes in Vδ2^+^ TCRδ and γ (*n* = 7). **f** CD27 and CD45RA T-cell memory marker expression by Vγ9^+^ Vδ2^+^ T cells from adult peripheral blood samples (*n* = 18). **g** Comparison of CD27 expression levels (MFI) on CD27^+^ CD45RA^neg^ Vγ9^+^ Vδ2^+^ T cells (*n* = 18), CD27^hi^ Vδ1^+^ (*n* = 14) and CD27^+^ CD45RA^+^ CD8^+^ T cells (*n* = 14) from adult peripheral blood samples. **h** Summary radar plot data detailing the mean % positive cells for each indicated T-cell marker analysed within each sub-population of Vγ9^+^ Vδ2^+^ (*n* = 18), Vδ1^+^ (*n* = 14) and CD8^+^ αβ T cells (*n* = 14). **i** Healthy adult peripheral blood donor 6 and 24’s expression of CD27 and CD45RA T-cell memory markers on Vγ9^+^ Vδ2^+^ T cells. Error bars indicate means ± SEM; *P < 0.05; **P < 0.01; ****P < 0.0001; *p*-values were determined by Student's *t*-test (**a**, **e**) and one-way ANOVA with Tukey’s post hoc testing (**g**)

Due to the largely similar Vδ2^+^ repertoire characteristics found in cord blood and adult, we next assessed the proportion of CDR3 amino acid sequences that were “public”, i.e., detected in more than one donor. Consistent with a high degree of diversity within the TCRδ repertoire, adult TCRδ2 repertoires contained few shared CDR3δ sequences (mean 8.7%), although this proportion was increased in cord blood CDR3δ repertoires (mean 36.8%; Fig. 2a, Supplementary Table 1 and 2). However, a mean 79.3% of the CDR3γ9 amino acid sequences present in a given individual were shared with at least one other adult or cord blood donor (Fig. 2b). Moreover, the majority of sequences in cord blood were directly shared in adult repertoires (Fig. 2c), indicating a core set of CDR3γ9 sequences in adult donors essentially indistinguishable from equivalent unrelated neonatal donor sequences.

To further explore this publicity, we examined the 10 most prevalent CDR3γ9 sequences from our cohort (see Methods), representing a mean 38.9% of the total CDR3γ9 repertoire (Fig. 2d). This highlighted a set of public CDR3γ9 sequences that were prevalent in both adult and cord blood repertoires, and at similar frequencies (Fig. 2d). Notably, two simple CDR3γ9 sequences were prevalent in all adult and neonatal repertoires analysed: CALWEVQELGKKIKVF (mean 13.1% in adult, and mean 24.6% in neonates) and CALWEVRELGKKIKVF (mean 4.3% in adults and mean 5.4% in neonates). Two processes explain the high degree of sharing within the CDR3γ9 repertoire. Firstly, single-cell TCR sequencing highlighted that CALWEVQELGKKIKVF could be generated by simple recombination of Vγ9 and JγP gene segments containing no added N nucleotides (“germline”) (Table 1). This sequence was common in foetal liver^30^, and foetal blood^25^ and observed to persist into adulthood^31^. While a high prevalence in repertoire data might indicate an expanded clonotype, single-cell TCR analysis also revealed that this germline-encoded CDR3γ9 sequence in fact was comprised of multiple TCR “pseudoclonotypes”, in which the same CDR3γ9 was independently generated and paired with different CDR3δ2 sequences in the same individual (Table 1). Secondly, CDR3γ9 sequences such as CALWEVQELGKKIKVF and CALWEVRELGKKIKVF could also be generated by convergent recombination, whereby variable degrees of exonuclease activity and N nucleotide addition result in the generation of the same CDR3γ amino acid sequences from different nucleotide sequences (Table 1). Both of these processes explain why CDR3γ9 repertoires were less diverse compared to CDR3δ repertoires (Fig. 2e), and are consistent with the generation of public TCR sequences^32^, but importantly, these mechanisms are not evident in Vδ1^+^ TCRγ repertoires^11^.

**Table 1:** Single-cell TCR sequencing of adult Vγ9^+^ Vδ2^+^ T cells

Donor	CDR3γ9 (amino acid)	CDR3γ9 nucleotide (nt) sequence	N nt	P nt	CDR3δ2 (amino acid)
3	CALW**EVQ**ELGKKIKVF	TGTGCCTTGTGGGAGGTGCAAGAGTTGGGCAAAAAAATCAAGGTATTT	Germline	0	CACDS**L**LGDTPNFDKLIF
	CALW**EVQ**ELGKKIKVF	TGTGCCTTGTGGGAGGTGCAAGAGTTGGGCAAAAAAATCAAGGTATTT	Germline	0	CACDT**G**GAQSWDTRQMFF
28	CALW**EVQ**ELGKKIKVF	TGTGCCTTGTGGGAGGT***T***CAAGAGTTGGGCAAAAAAATCAAGGTATTT	1	0	CACDS**L**GTYTDKLIF
	CALW**EVQ**ELGKKIKVF	TGTGCCTTGTGGGAGGTGCAAGAGTTGGGCAAAAAAATCAAGGTATTT	Germline	0	CACDI**I**LGGQYTDKLIF
35	CALW**EVQ**ELGKKIKVF	TGTGCCTTGTGGGAGGTGCA***G***GAGTTGGGCAAAAAAATCAAGGTATTT	1	2	CACES**L**GPTGGNPSSDKLIF
	CALW**EVQ**ELGKKIKVF	TGTGCCTTGTGGGAGGT***T***CAAGAGTTGGGCAAAAAAATCAAGGTATTT	1	0	CACDT**I**THRTGGPQVTDKLIF
	CALW**EVQ**ELGKKIKVF	TGTGCCTTGTGGGAGGTGCAAGAGTTGGGCAAAAAAATCAAGGTATTT	Germline	0	CACDG**L**GGEYTDKLIF
42	CALW**EVQ**ELGKKIKVF	TGTGCCTTGTGGGAGGTGCAAGAGTTGGGCAAAAAAATCAAGGTATTT	Germline	0	CACDK**V**GYGSPWDTRQMFF
	CALW**EVQ**ELGKKIKVF	TGTGCCTTGTGGGAGGTGCAAGAGTTGGGCAAAAAAATCAAGGTATTT	Germline	0	CACDT**I**PTGGHDPYTDKLIF
231	CALW**EVQ**ELGKKIKVF	TGTGCCTTGTGGGAGGT***A***CAAGAGTTGGGCAAAAAAATCAAGGTATTT	1	0	CACDT**V**TRDKGADKLIF
	CALW**EVQ**ELGKKIKVF	TGTGCCTTGTGGGAGGT***A***CAAGAGTTGGGCAAAAAAATCAAGGTATTT	1	0	CACDT**M**GARHTDKLIF
	CALW**EVQ**ELGKKIKVF	TGTGCCTTGTGGGAGGT***A***CAAGAGTTGGGCAAAAAAATCAAGGTATTT	1	0	CACDT**G**FGLVQSHGPKRTDKLIF
	CALW**EVQ**ELGKKIKVF	TGTGCCTTGTGGGAGGTGCAAGAGTTGGGCAAAAAAATCAAGGTATTT	Germline	0	CACDR**L**GGNTDKLIF
1014	CALW**EVQ**ELGKKIKVF	TGTGCCTTGTGGGAGGTGCAAGAGTTGGGCAAAAAAATCAAGGTATTT	Germline	0	CACDT**V**PSTGGPKDTDKLIF
	CALW**EVQ**ELGKKIKVF	TGTGCCTTGTGGGAGGTGCAAGAGTTGGGCAAAAAAATCAAGGTATTT	Germline	0	CACDT**G**SLQRYNSWDTRQMFF
261	CALW**EVR**ELGKKIKVF	TGTGCCTTGTGGGAGGT***CAG***AGAGTTGGGCAAAAAAATCAAGGTATTT	3	0	CACDT**W**GTVGRTDKLIF
	CALW**EVR**ELGKKIKVF	TGTGCCTTGTGGGAGGTGC***G***AGAGTTGGGCAAAAAAATCAAGGTATTT	1	1	CACDT**V**GDWGTPLYTDKLIF

Public TCRγ9 clonotypes and their paired TCRδ2 sequence from seven donors. Sequences were analysed using IMGT Junction Analysis, which identified V, D and J gene segments used and highlighted N nucleotide addition (bold and italic) and P nucleotide addition (underlined). The table depicts CDR3γ9 amino acid sequence, CDR3γ9 nucelotide (nt) sequence, germline or N nt addition, P nt addition, CDR3δ2 amino acid sequence. Hydrophobic amino acids at position 5 (see Methods section) in the CDR3δ2 sequence are highlighted in bold

### Altered Vγ9^+^ Vδ2^+^ T-cell phenotype links to clonal expansion

We evaluated the memory phenotype of Vδ2^+^ T cells from each donor to establish the relationship with TCR clonotype. Adult Vγ9^+^ Vδ2^+^ T cells from healthy donors (16/18 of total donors, 5/7 TCR repertoire study donors) regularly display a major CD27^+^ CD45RA^−^ phenotype (similar to Central Memory (CM) CD8^+^ αβ T cells) and a minor CD27^−^ CD45RA^−^ phenotype (similar to Effector Memory (EM) CD8^+^ αβ T cells) (Fig. 2f), consistent with other studies^33^. Importantly, Vγ9^+^ Vδ2^+^ T cells differ from Vδ1^+^ T-cell populations in their expression levels of CD27 (Fig. 2g) and distinct naive and effector T-cell surface marker expression (Fig. 2h).

Although overall our adult cohort displayed similar Vγ9^+^ Vδ2^+^ TCR diversity to cord blood samples, two adult donors (6 and 24) appeared to exhibit a degree of clonotypic focusing, based on their low D75 values for both TCRγ (mean 2.9) and TCRδ (mean 5.0) combined with their higher cumulative frequency of the 10 most prevalent clonotypes (mean 60.5% of TCRγ and mean 50.3% of TCRδ) (Fig. 1a). Consistent with this, each donor displayed individual clonotypes with a similar amplified frequency in both TCR γ and δ repertoires (Fig. 1a). Both donor 6 and 24 each possessed a distinct uncommon but shared sequence, CALWEVRKELGKKIKVF (detected in donor 6 and also in 2 other donors) or CALWEKMQELGKKIKVF (detected in donor 24 and in 4 other donors), usually outside the top 10 most prevalent shared clonotypes, but now representing 18.5% (donor 6) and 44.6% (donor 24) of the TCRγ9 repertoire, respectively (Fig. 2d). Also, donor 6 possessed a common sequence found in all donors, CALWETQELGKKIKVF, normally accounting for a mean 0.95% of the TCRγ9 repertoire, but increased to 22.9% of donor 6’s TCRγ9 repertoire (Fig. 2d). Furthermore, these unusual clonal amplifications were linked to phenotypic changes in the total Vγ9^+^ Vδ2^+^ T-cell population, whereby these donor’s cells adopted a predominant CD27^−^ CD45RA^−^ phenotype (Fig. 2i). These data suggest that despite the public repertoire and associated dominant phenotype (CD27^+^ CD45RA^−^) in the majority of donors, there is scope within the Vγ9^+^ Vδ2^+^ TCR repertoire for a degree of specific clonal amplification, alongside concomitant phenotypic changes.

### A discrete Vγ9^−^ Vδ2^+^ subset persists from birth into adults

**Fig. 3:**
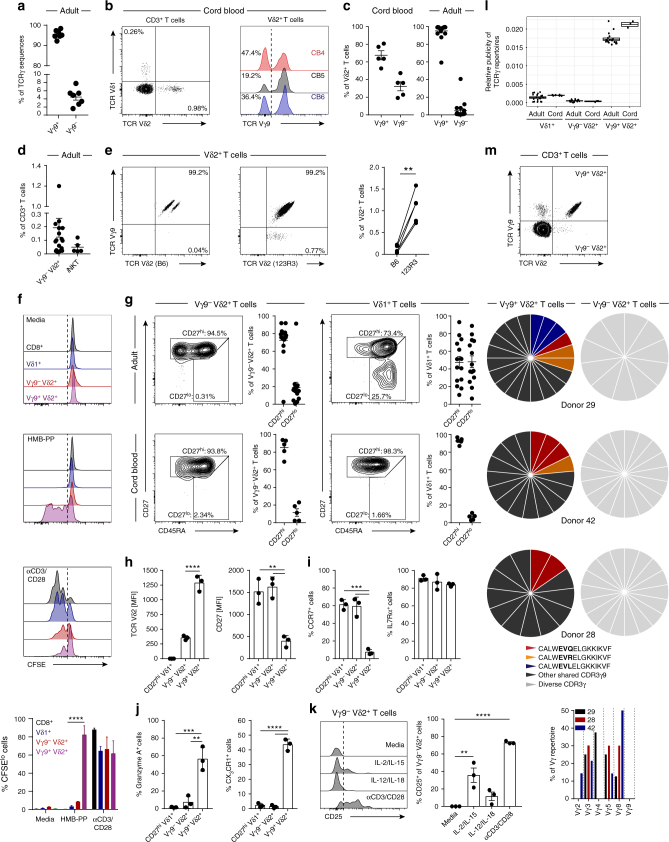
Vγ9^−^ Vδ2^+^ T cells are clonally and phenotypically distinct from Vγ9^+^ Vδ2^+^ T cells. **a** Frequency of Vγ9 chain usage in Vδ2^+^ TCR repertoire sequencing data from adult peripheral blood (*n* = 7). **b** Identification of Vδ2^+^ T cells in CD3^+^ T cells (left) and Vγ9^−^ cells within Vδ2^+^ T cells (right) from cord blood (*n* = 5). **c** Frequency of Vγ9^−^ and Vγ9^+^ cells in Vδ2^+^ T cells from cord blood (left; *n* = 5) and adult peripheral blood (right; *n* = 18). **d** Frequency of Vγ9^−^ Vδ2^+^ T cells (*n* = 18) and NKT cells (αGalcer/CD1d^+^; *n* = 5) in adult peripheral blood. **e** Vγ9^−^ Vδ2^+^ T cells identified by TCR Vδ2 antibody clones (B6 and 123R3) in matched adult peripheral blood donors (*n* = 5). **f** Proliferation by CFSE dilution of CD8^+^ αβ and γδ T-cell populations from PBMC treated with medium alone, 10 nM HMB-PP or 5 μg/ml anti-CD3/CD28 (*n* = 5). **g** CD27 and CD45RA T-cell memory marker expression profiles on Vγ9^−^ Vδ2^+^ and Vδ1^+^ T cells from adult peripheral blood (top row: Vδ2, *n* = 14; Vδ1, *n* = 14) and cord blood (bottom row: Vδ2 and Vδ1, *n* = 5). **h** Vδ2^+^ TCR and CD27 expression levels, **i** naive and **j** effector T-cell marker expression on donor matched γδ T-cell populations from adult peripheral blood (*n* = 3). **k** Sorted CD3^+^ T cells were incubated for 72 h with cytokines or anti-CD3/CD28 beads. Vγ9^−^ Vδ2^+^ T cells were then assessed for the upregulation of CD25 (*n* = 3). **l** Comparison of CDR3γ sequence sharing in γδ T-cell repertoires from adult peripheral blood (Vδ1, *n* = 20; Vδ2, *n* = 7) and cord blood (Vδ1, *n* = 5; Vδ2, *n* = 3). **m** CDR3γ sequence analysis of single cell sorted Vγ9^+^ and Vγ9^−^ Vδ2^+^ T cells, public sequences are coloured (black, shared sequences from deep sequencing); graph shows Vγ usage of Vγ9^−^ Vδ2^+^ TCR sequences from each donor. Error bars indicate means ± SEM; **P < 0.01; ***P < 0.001; ****P < 0.0001; *p*-values were determined by paired *t*-test (**e**), RM two-way ANOVA with Tukey’s post hoc testing (**f**); one-way ANOVA with Tukey’s post hoc testing (**h**, **i**) or Dunnett’s post hoc testing (**j**, **k**)

TCR γ repertoire datasets from Vδ2^+^ T cells were dominated by Vγ9 TCR sequences, but we noted the presence of non-Vγ9 (i.e., Vγ2–8) sequences in adult TCRγ repertoires (mean 4.46%) (Fig. 3a). Although such chain usage could conceivably represent second productive TCRγ rearrangements in Vγ9^+^ Vδ2^+^ T cells, an alternative possibility was that they reflected the existence of an unusual Vγ9^−^ Vδ2^+^ T-cell subset that may persist in adulthood. As Vγ9^−^ TCR usage by neonatal Vδ2^+^ T cells has been previously reported^34^, we established flow cytometry-based identification of Vγ9^−^ cells in cord blood Vδ2^+^ T cells (Fig. 3b) and quantified their relative frequency (mean 32.41%) (Fig. 3c). Importantly, adult Vδ2^+^ T-cell pools retained this Vγ9^−^ subset at low frequency (mean 4.82%) (Fig. 3c and Supplementary Fig. 2a), but on average occupying a similar frequency within total peripheral blood T cells to that of α-GalCer/CD1d reactive natural killer T cells (NKT) (Fig. 3d and Supplementary Fig. 2b). Notably, the identification of Vγ9^−^Vδ2^+^ T cells was only possible with the use of an anti-Vδ2 TCR antibody clone, 123R3 (Miltenyi), while this population was undetectable using another TCR Vδ2 antibody clone, B6 (Biolegend), which is likely to be specific for the Vγ9^+^ Vδ2^+^ TCR pairing (Fig. 3e).

### Vγ9^−^ Vδ2^+^ T cells and Vγ9^+^ Vδ2^+^ T cells are distinct

As adult peripheral blood Vδ2^+^ T cells are commonly responsive to host and microbial P-Ags, we assessed the proliferative capacity of Vγ9^−^ Vδ2^+^ T cells towards microbial P-Ag (HMB-PP). While CD8^+^, both Vδ2^+^ subsets and Vδ1^+^ T cells all proliferated in response to anti-CD3/CD28 stimulation, only Vγ9^+^ Vδ2^+^ T cells responded to HMB-PP (Fig. 3f). We then compared the memory phenotype of Vγ9^+^ Vδ2^+^ T cells to other human γδ T-cell populations. Cord blood Vγ9^−^ Vδ2^+^ T cells expressed a CD27^hi^ phenotype which extended to the adult population, remarkably similar to naive-like Vδ1^+^ T cells (Fig. 3g). We next assessed the expression of naive and effector T-cell markers in paired γδ T-cell populations from the same donors. Vγ9^−^ Vδ2^+^ T cells commonly displayed lower levels of TCR Vδ2 expression and higher levels of CD27 expression than their Vγ9^+^ counterparts (Fig. 3h). Vγ9^−^ Vδ2^+^ T cells expressed the lymphoid homing receptor CCR7, the homoeostatic cytokine receptor IL7Rα (Fig. 3i) but lacked the cytotoxic effector molecule Granzyme (Grz) A or the endothelial homing receptor CX_3_CR1 (Fig. 3j), with these expression patterns contrasting with Vγ9^+^ Vδ2^+^ T cells (Vδ2^hi^ CD27^int^ CCR7^−^ IL7Rα^+^ Grz A^+^ CX_3_CR1^+^) but together highly similar to CD27^hi^ Vδ1^+^ T cells (Fig. 3h–j). Moreover, Vγ9^−^ Vδ2^+^ T cells were responsive to CD3/CD28 stimulation, but like Vδ1 T cells were unresponsive to IL-12/IL-18 (Fig. 3k), whereas Vγ9^+^ Vδ2^+^ T cells were responsive to both CD3/CD28 and IL-12/IL-18^11^. CD27^hi^ Vδ1^+^ T-cell populations lack expanded clonotypes and possess a diverse and private TCR repertoire^11^. Analysis of the publicity of adult Vγ9^−^ Vδ2^+^ TCR repertoires indicated a far more private TCRγ repertoire compared to Vγ9^+^ Vδ2^+^ T cells (Fig. 3l), but equivalent to the highly private Vδ1^+^ TCR repertoire^11^. This was confirmed by single-cell TCR sequencing of adult Vγ9^−^ Vδ2^+^ T-cell populations, which contained diverse TCR sequences, comprising a range of Vγ chains (Fig. 3m), and featuring TCR γ and δ chains that each lacked motifs previously linked to P-Ag reactivity (Table 2). In contrast, Vγ9^+^ Vδ2^+^ T cells sorted from the same donors displayed sequences that included prevalent shared TCRγ sequences (Fig. 3m and Table 1). Collectively, these data suggest that the Vγ9^−^ Vδ2^+^ subset is functionally, phenotypically and clonotypically distinct from its Vγ9^+^ counterparts, but may share a similar biology to that of Vδ1^+^ T cells.

**Table 2:** Single-cell TCR sequencing of Vγ9^−^ Vδ2^+^ T cells

Donor	CDR3δ2	Dδ	Jδ	CDR3δ length	Vγ	Jγ	CDR3γ length
3	CACGS**W**WGTYTDKLIF	1,3	1	14	8	P1	11
231	CACDT**I**VGDTLTDKLIF	3	1	15	8	P1	9
	CACVR**G**GGYPRGADKLIF	3	1	16	8	1/2	14
	CACDT**E**GEVNTDKLIF	3	1	14	4	P1	11
261	CACDT**R**FRPGRSARVVRLTAQLFF	1	2	22	3	P1	14
Liver 1	CACSR**E**QAHTDKLIF	3	1	13	3	1/2	10
	CACDT**G**WGIRGRYTDKLIF	3	1	17	4	1/2	11
Liver 2	CACDR**S**GARWDKLIF	—	1	13	5	1/2	10
42	CACDA**R**HYWGISTDKLIF	3	1	16	2	1/2	12
	CACDA**R**LGEHTDKLIF	3	1	14	ND	ND	ND
	CACDR**M**GDLPSSWDTRQMFF	3	3	18	3	1/2	13
	CACGS**S**WGVSHYTDKLIF	3	1	16	5	1/2	10
	CACDL**L**GDFRTDKLIF	3	1	14	8	1/2	8
	CACSV**R**GHWGRSTDKLIF	3	1	16	8	1/2	14
	CACDT**R**GIGDTLPDKLIF	3	1	16	8	P1	12
	CACAH**G**YWGTPWDTDKLIF	3	1	17	3	P1	11
	CACDT**G**DTDTDKLIF	3	1	13	8	P1	13
	CACDR**L**LLGDTDKLIF	3	1	14	ND	ND	ND
	CACDT**R**RTGGRDKLIF	3	1	14	5	1/2	9
	CACDK**R**IRWGNPYTDKLIF	3	1	17	8	P2	10
	CACEV**P**SYEPYWGTKKYTDKLIF	2,3	1	21	3	1/2	11
	CACDT**G**DWGINTDKLIF	3	1	15	8	P1	14
	CACDQ**K**YWGGSSTDKLIF	3	1	16	2	P	13

TCRδ sequences of clonally expanded Vγ9^−^ Vδ2^+^ TCRs from donors expressing a CD27^lo/neg^ phenotype (top section). All TCRδ2 sequences from a representative healthy donor with a CD27^hi^ phenotype (bottom section). Sequences were analysed using IMGT Junction Analysis, which identified V, D and J gene segments used. The table depicts CDR3δ sequences, Dδ usage, Jδ usage, CDR3 length, Vγ usage, Jγ usage and CDR3γ length. Amino acids (aa) at position 5 of the CDR3 sequence (see Methods) are highlighted in bold. ND not determined

### Vγ9^−^ Vδ2^+^ T cells clonally expand into effectors

**Fig. 4:**
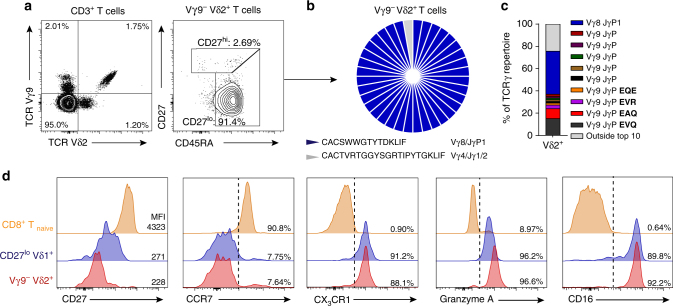
Vγ9^−^ Vδ2^+^ T cells undergo clonal expansion. **a** Identification of Vγ9^−^ Vδ2^+^ T cells (left) and CD27/CD45RA T-cell memory marker expression on Vγ9^−^ Vδ2^+^ T cells (right) in a peripheral blood sample from “donor X”. **b** Single-cell CDR3δ sequence analysis and Vγ usage by Vγ9^−^ Vδ2^+^ T cells sorted from a peripheral blood sample from “donor X”. **c** TCRγ repertoire analysis of Vδ2^+^ T cells from “donor X”, highlighting the frequency of “donor X’s” Vγ9^−^ Vδ2^+^ T-cell clone (from **b**) within the top 10 most prevalent clonotypes, highlighted are prevalent Vγ9^+^ clonotypes (EVQ, EAQ, EVR and EQE). **d** Analysis of naive and effector T-cell marker expression by Vγ9^−^ Vδ2^+^, CD8^+^ CD27^hi^ CD45RA^hi^ (CD8^+^ T_naive_) and CD27^lo/neg^ Vδ1^+^ T-cell populations from a peripheral blood sample from “donor X”

Within our healthy donor cohort, one individual (“donor X”) had a substantially increased Vγ9^−^ Vδ2^+^ T-cell population, comprising 1.2% of all CD3^+^ T cells (Fig. 4a), far higher than the 17 other healthy donors (mean 0.13% of CD3^+^ T cells). Importantly, and in contrast to the dominant CD27^hi^ phenotype displayed by Vγ9^−^ Vδ2^+^ T cells in all other healthy donors, “donor X’s” Vγ9^−^ Vδ2^+^ T-cell population had downregulated CD27 and retained CD45RA expression (CD27^lo/neg^ phenotype), now accounting for 91.4% of the Vγ9^−^ Vδ2^+^ T-cell population (Fig. 4a). We next carried out single-cell TCR sequencing to determine if any particular γδ TCR clonotypes correlated with this phenotypic change. Strikingly, a single clone now dominated the Vγ9^−^ Vδ2^+^ T-cell population (35/36 single cells sequenced), composed of a Vδ2 CDR3 (CACGSWWGTYTDKLIF) paired with a Vγ8–JγP1 chain (Fig. 4b), and now represented the single most dominant clonotype within the Vδ2^+^ T-cell repertoire (Fig. 4c). As this dominant clonotype occurred in conjunction with a CD27^lo/neg^ phenotype, we assessed effector T-cell marker expression. “Donor X’s” Vγ9^−^ Vδ2^+^ T-cell population expressed the antibody-mediated cytotoxicity receptor CD16 (FcγRIIIa), CX_3_CR1, Grz A and had downregulated CCR7, in contrast to naive CD8 T cells, but closely matching CD27^lo/neg^ Vδ1^+^ T cells (Fig. 4d), which also comprised dominant clonotypes^11^. These results highlight the potential for the Vγ9^−^ Vδ2^+^ T-cell compartment to undergo clonal selection and differentiation, mimicking the transition to clonality and effector status previously observed in Vδ1^+^ T cells^11^.

### Clonally focused Vγ9^−^ Vδ2^+^ T cells infiltrate human liver

**Fig. 5:**
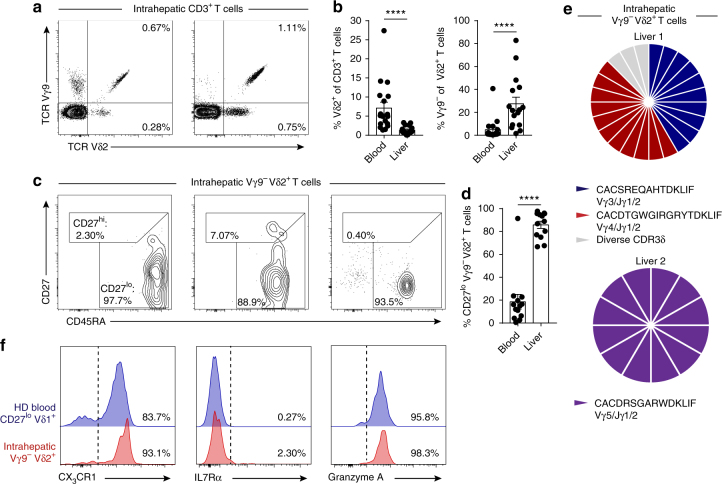
Clonally expanded Vγ9^−^ Vδ2^+^ T cells infiltrate human liver tissue. **a** Representative identification of intrahepatic Vγ9^−^ Vδ2^+^ T cells in CD3^+^ lymphocytes obtained from liver tissue and **b** summary data of the frequency of Vδ2^+^ T cells in CD3^+^ T cells (left) or Vγ9^−^ Vδ2^+^ T cells in Vδ2^+^ T cells (right) from peripheral blood (*n* = 18) and liver tissue (*n* = 16). **c** Representative intrahepatic Vγ9^−^ Vδ2^+^ T-cell expression profiles of CD27 and CD45RA T-cell memory markers and **d** summary data from peripheral blood (*n* = 18) and liver tissue (*n* = 16). **e** Single-cell CDR3δ sequence analysis and Vγ usage by intrahepatic Vγ9^−^ Vδ2^+^ T cells sorted from two independent liver tissue samples. **f** Representative analysis of CX_3_CR1, IL7Rα and Granzyme A expression by intrahepatic Vγ9^−^ Vδ2^+^ T cells (*n* = 3) and a healthy donor’s (HD) CD27^lo/neg^ Vδ1^+^ T-cell population (*n* = 8). Error bars indicate means ± SEM; ****P < 0.0001; *p*-values were determined by Mann–Whitney *t*-test (**b**, **d**)

Vδ1^+^ T cells are predominantly associated with a role in tissue immunity^35,36^. To address whether the Vγ9^−^ Vδ2^+^ T-cell population was also represented in solid tissues, we analysed human liver samples for the presence of intrahepatic Vγ9^−^ Vδ2^+^ T cells (Fig. 5a). While the Vδ2^+^ T cells in the liver comprised a lower proportion of total CD3^+^ T cells than in peripheral blood, intrahepatic Vδ2^+^ T cells generally contained a higher fraction of Vγ9^−^ cells (Fig. 5b). Phenotypic analysis of intrahepatic Vγ9^−^ Vδ2^+^ T cells revealed these cells to consistently display a CD27^lo/neg^ phenotype (Fig. 5c, d). Single-cell TCR sequencing analysis, highlighted that a majority of intrahepatic Vγ9^−^ Vδ2^+^ T cells were comprised of a limited set of expanded clonotypes (Fig. 5e and Table 2). Moreover, clonally expanded intrahepatic Vγ9^−^ Vδ2^+^ T cells expressed effector markers CX_3_CR1 and Grz A but had downregulated IL7Rα (Fig. 5f).

### Clonal expansion of Vγ9^−^ Vδ2^+^ T cells in acute CMV infection

**Fig. 6:**
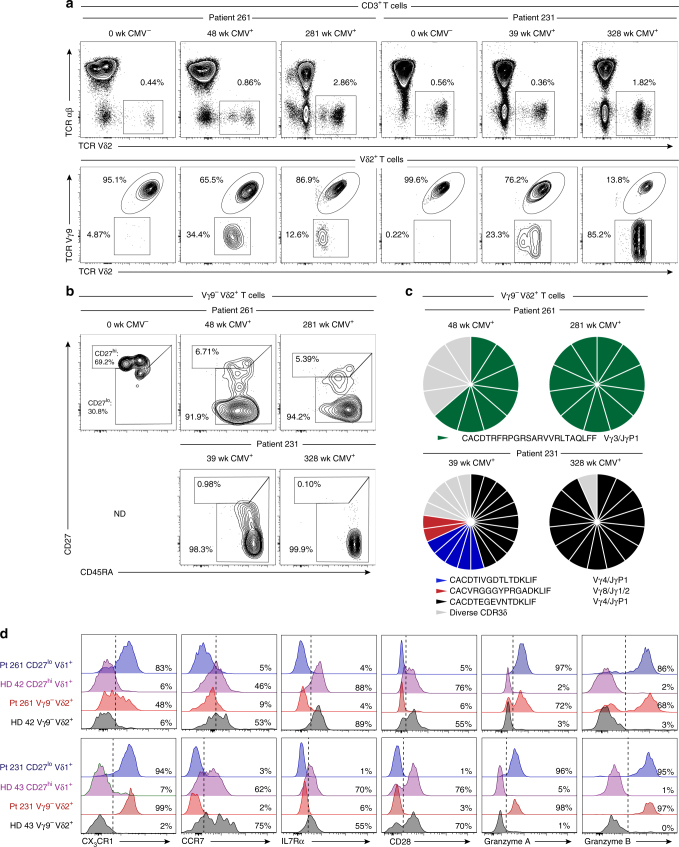
Vγ9^−^ Vδ2^+^ T cells exhibit TCR-specific clonal expansion in response to viral infection. **a** Identification of Vδ2^+^ T cells in CD3^+^ T cells (top row) and Vγ9^−^ cells within Vδ2^+^ T cells (bottom row) in longitudinal peripheral blood samples from two CMV-seronegative kidney transplant patients developing post-operative acute CMV infection (CMV-seroconversion: patient 231, 8 weeks; patient 261, 7 weeks). **b** CD27 and CD45RA T-cell memory marker expression profiles by detectable Vγ9^−^ Vδ2^+^ T cells populations in longitudinal peripheral blood samples from patients 231 and 261. Cytometry data from patient 261 at 281 weeks was acquired on an alternative flow cytometer (**a** and **b**). **c** Single-cell CDR3δ sequence analysis and Vγ usage by Vγ9^−^ Vδ2^+^ T cells sorted from patients 231 and 261 peripheral blood samples between 39 and 328 weeks after transplantation. **d** Analysis of the indicated naive and effector T-cell markers by Vγ9^−^ Vδ2^+^ T cells from patients 261 (week 48) and 231 (week 328) and in comparison with CD27^hi^, CD27^lo/neg^ Vδ1^+^ and Vγ9^−^ Vδ2^+^ T cells from the peripheral of blood of two healthy donors analysed in parallel. ND no detectable population

The clonotypic and phenotypic parallels between Vγ9^−^ Vδ2^+^ and Vδ1^+^ γδ T cells, combined with their strong distinction from P-Ag sensing Vγ9^+^ Vδ2^+^ T cells, prompted us to investigate if this subset was reactive to acute cytomegalovirus (CMV) infection, previously shown to drive the numeric^37^ and clonal^12^ expansion of Vδ1^+^ T cells. We examined a cohort of five CMV-seronegative (CMV^−^) patients receiving CMV-seropositive (CMV^+^) kidney transplants who then went on to either develop post-operative acute CMV infection (patient 261, 231 and 1014), or who remained CMV^−^ (patient 279 and 282)^38^. We analysed peripheral blood samples for Vγ9^−^ Vδ2^+^ T cells before and after post-transplant CMV infection. Prior to CMV infection, at 0–4 weeks post-transplant, Vγ9^−^ Vδ2^+^ T cells represented low frequency populations in all donors (Fig. 6a and Supplementary Fig. 3). However, following CMV infection, occurring between 5–8 weeks post-transplant, we observed the substantial expansion of the Vγ9^−^ Vδ2^+^ T-cell population in donor 261 and 231 (Fig. 6a). Conversely, patients 282 and 279 who did not become infected with CMV, displayed no expansion of Vγ9^−^ Vδ2^+^ T cells over a 5-year period after transplantation (Supplementary Fig. 3a). Of note is patient 1014, who became infected with CMV but did not display an expanded Vγ9^−^ Vδ2^+^ T-cell population (Supplementary Fig. 3b), suggesting inter-individual differences in the Vγ9^−^ Vδ2^+^ T-cell response to CMV infection consistent with observations in Vδ1^+^ T cells from persistently infected CMV^+^ healthy adult donors^11^. Phenotypic analysis of Vγ9^−^ Vδ2^+^ T cells from CMV^-^ patients 279 (Supplementary Fig. 3c) and 261 immediately after transplant (Fig. 6b), indicated a CD27^hi^ phenotype. Conversely after CMV infection, the expanded Vγ9^−^ Vδ2^+^ T-cell populations in patients 261 and 231 acquired a dominant CD27^lo/neg^ phenotype (Fig. 6b). This prompted us to perform single-cell TCR sequencing of expanded Vγ9^−^ Vδ2^+^ T-cell populations in donors 261 and 231, which identified the selective expansion of dominant clonotypes after CMV infection and evidence of continued clonotypic selection 5 years later (Fig. 6c and Table 2). Finally, we assessed functional T-cell marker expression in Vγ9^−^ Vδ2^+^ T cells from patient 261 and 231 following CMV infection. Vγ9^−^ Vδ2^+^ T cells from healthy donors expressed CCR7, IL7Rα and the co-stimulatory molecule CD28, while patient 261 and 231’s Vγ9^−^ Vδ2^+^ T cells had downregulated these markers and upregulated CX_3_CR1 and Grz A and B (Fig. 6d). Expression of these effector markers occurred concurrently with Vγ9^−^ Vδ2^+^ T-cell clonal expansion at ~1 year (patient 261) and were stable for over 5 years (patient 231) (Fig. 6c, d). These data reveal that as for Vδ1^+^ T cells, Vγ9^−^ Vδ2^+^ T cells exhibit a strong relationship between clonotype and phenotype and are suggestive of a role in adaptive antiviral immunosurveillance.

## Discussion

This study sheds light on the biology of Vδ2^+^ T cells, highlighting divergent Vγ9^+^ and Vγ9^−^ subsets. Despite the ~ten-fold increase in Vγ9^+^ Vδ2^+^ T-cell numbers in the first year of life, our data indicate that the extent of clonotypic focusing within the Vδ2-associated Vγ9^+^ repertoire is broadly similar between neonatal cord blood and adulthood. This finding contrasts with the Vδ1^+^ TCR repertoire, which typically displays pronounced clonotypic focusing in adults relative to a highly unfocused neonatal repertoire^11^. In addition, our analyses reveal an underappreciated degree of public amino acid sequences in the Vγ9 repertoire. This degree of Vγ publicity, which relates to both recombination of simple Vγ9 sequences (either germline encoded or with limited N nucleotide addition) and convergent recombination, underlines the semi-invariant nature of the Vγ9^+^ Vδ2^+^ TCR repertoire. Importantly, using single-cell approaches public Vγ clonotypes were shown frequently to pair with diverse Vδ2 chains within single individuals, and, therefore, do not usually represent expanded clonotypes. The innate-like biology of the semi-invariant Vγ9^+^ Vδ2^+^ T-cell subset contrasts markedly with the Vδ1^+^ subset, which displays clear hallmarks of adaptive immunity such as clonal amplification and differentiation from an initially unfocused, private TCR repertoire.

Collectively, our repertoire data suggest a model involving a multi-layered selection of Vγ9^+^ Vδ2^+^ T cells from foetal development into adulthood. First, as previously suggested by Dimova et al.^25^, we propose gestational selection of the Vγ9^+^ Vδ2^+^ subset, potentially via exposure to endogenous P-Ag (e.g., IPP) most likely mediated by BTN3A1^15–17,39,40^, allowing pre-programming of a P-Ag reactive Vγ9^+^ Vδ2^+^ TCR repertoire. In keeping with this, we observed that Vγ9 chains are heavily enriched for the JγP segment, which is not generally present in Vγ9 chains present in Vδ1^+^ TCR repertoires^11^; similarly the Jδ1^+^ CDR3δ2 repertoire in cord blood is enriched for a hydrophobic amino acid at position 5 required for P-Ag recognition^21,29^, but not in Vδ2 sequences in Vγ9^−^ Vδ2^+^ T cells (see below). Second, in the context of the reported lack of thymic output of Vδ2^+^ T cells after birth^41^, our observation that adult and cord blood repertoires contain overlapping canonical P-Ag-sensing Vγ9 sequences strongly indicates there is polyclonal post-natal expansion of this pre-selected repertoire, most likely in response to microbial P-Ag exposure^11^. However, our observation that cord blood Vδ2 sequences generally utilised Jδ3 segments (also observed in foetal γδ T cells,^42^), whereas adult Vδ2 sequences preferentially used Jδ1 segments, suggests that post-natal microbial exposure may impose some constraints on the TCR repertoire that differ from those required in gestation. Of note, Vδ2–Jδ1 sequences typically contain a hydrophobic amino acid previously highlighted as important for Vγ9^+^ Vδ2^+^ T-cell reactivity to microbially-derived P-Ag^21,29^ whereas this is less conserved in Vδ2–Jδ3. This might imply Vγ9^+^ Vδ2^+^ T cells bearing Jδ1 clonotypes preferentially respond to microbial P-Ag after birth compared to Jδ3 clonotypes, a possibility which future studies can address. Finally, although most individuals’ Vγ9^+^ Vδ2^+^ T cells were dominated by public Vγ9 sequences and associated with a common central memory phenotype, our observation that a minority of individuals exhibit amplification of unusual public clonotypes, concomitant with adoption of an effector memory phenotype, most likely reflects a third level of selection that imparts a degree of plasticity in this fundamentally innate-like paradigm. This observation extends previous work by Ryan et al.^33^ indicating that further functional and transcriptional changes are linked to an effector memory phenotype in Vγ9^+^ Vδ2^+^ T cells. Therefore, the semi-invariant Vγ9^+^ Vδ2^+^ repertoire may provide scope for selective TCR-mediated responses to microbial P-Ag. Importantly, our model shares a number of features with MAIT cells, a semi-invariant population that is also subject to both gestational selection (through interactions with MR1) and post-natal expansion in response to microbial infection/colonisation^43^, and that displays clonotype-specific responses to metabolite ligands^44^ and microbial species^45^.

Our analyses also highlight a population of Vγ9^−^ Vδ2^+^ T cells that is highly distinct from the Vγ9^+^ Vδ2^+^ P-Ag-reactive subset, and present in all adult peripheral blood and cord blood, where they commonly exist as a CD27^hi^, TCR diverse subset phenotypically equivalent to naive-like Vδ1^+^ T cells. We note this distinction based on Vγ9 chain usage cannot be applied to the γδ T-cell compartment as a whole, as the Vδ1^+^ T-cell subset, which appears to exhibit an adaptive immunobiology, contains a sizeable portion of Vγ9^+^ T cells^11^. Moreover, consistent with a distinct biology for Vγ9 chains in these two contexts, the Vγ9 CDR3 sequences associated with Vδ1 chains did not utilise JγP sequences and were highly distinct from those associated with Vδ2 chains^11^. Consistent with expression of TCRγ and TCRδ sequences divergent from the Vγ9^+^ Vδ2^+^ subset, the Vγ9^−^ Vδ2^+^ population was not P-Ag responsive. Thus, Vγ9^−^ Vδ2^+^ T cells represent a universal γδ subset that is clonotypically, phenotypically and functionally divergent from Vγ9^+^ Vδ2^+^ T cells. Moreover, as evidenced from a single unusual peripheral blood donor and several liver samples, Vγ9^−^ Vδ2^+^ T cells appeared capable of undergoing an adaptive-like programme of highly focused clonotypic expansion and concomitant phenotypic differentiation that closely matches that of Vδ1^+^ T cells but is not observed in Vγ9^+^ Vδ2^+^ T cells. This considerably extends the findings of Ravens et al.^12^, who detected unusual Vγ9^−^ Vδ2^+^ clonotypes in the peripheral blood of one healthy donor and one HSCT recipient.

This adaptive biology was confirmed by our analysis of patients following acute CMV infection, which highlighted a striking expansion of the Vγ9^−^ Vδ2^+^ subset after infection, linked to the selection of dominant clonotypes, which occurred concomitantly with differentiation from a naive CD27^hi^ to a CD27^lo/neg^ phenotype, and acquisition of CX_3_CR1 and cytotoxic granzymes, similar to CMV-specific cytotoxic CD8^+^ T cells^46,47^. Interestingly, the single healthy donor who exhibited a clonally expanded Vγ9^−^ Vδ2^+^ T-cell subset (“donor X”) was CMV^+^ and unusually, displayed elevated IgG titres, raising the possibility of recent CMV reactivation. Our findings therefore suggest a role for the Vγ9^−^ Vδ2^+^ T-cell subset in unconventional immunosurveillance against viral infection, and provide a clear indication that this subset assumes an adaptive immunobiology, highly similar to that suggested for Vδ1^+^ T cells^11,12^. These data suggest clonal selection from a CCR7^+^, potentially lymphoid tissue homing, naive-like Vγ9^−^Vδ2^+^ T-cell population that expresses a pool of TCRs, enabling amplification of responses to microbial challenges, via differentiation of cells bearing biologically relevant clonotypes to an effector phenotype, resulting in long-lived, functional γδ T-cell memory. The liver provided an attractive human model in which to examine Vγ9^−^ Vδ2^+^ T cells in solid tissues, as it functions as an important site for clearance of both commensals and pathogens from portal circulation, and has a high proportion of unconventional T cells, such as MAIT cells^48^. Our finding that clonally expanded effector Vγ9^−^ Vδ2^+^ T cells can be recruited to the liver suggests this subset may complement the innate-like recognition provided by such hepatic semi-invariant T cells with an unconventional adaptive memory response. Unfortunately, we were unable to obtain CMV serostatus for the patients from whom explanted liver samples were derived, and therefore unable to conclude if the presence of such hepatic Vγ9^−^ Vδ2^+^ T cells solely reflected CMV seropositivity or alternatively a wider set of immune challenges. Of relevance, a monoclonally expanded myocytotoxic T-cell population bearing a Vγ3^+^ Vδ2^+^ TCR detected in a patient with polymyositis highlights the potential of the Vγ9^−^ Vδ2^+^ T-cell subset to trigger autoimmunity^49,50^. Further studies are required to define additional microbial and non-microbial challenges that stimulate this unconventional T-cell subset.

In addition to clonal expansion in response to CMV, the finding that peripheral blood Vγ9^−^ Vδ2^+^ T cells were TCR responsive but unresponsive to IL-12/IL-18 is another feature that distinguishes them from Vγ9^+^ Vδ2^+^ T cells, and one that again aligns them closely with the Vδ1^+^ T-cell subset, which has also been highlighted to display adaptive features^11,12^ Further studies on such adaptive subsets (Vδ1^+^ and Vγ9^−^ Vδ2^+^ T cells), ideally ultimately in the context of physiological TCR ligands, are required to explore the full range of functional differences with innate-like subsets. Of note, Vγ9^+^ Vδ2^+^ T cells have previously been demonstrated to exert pleiotropic effector functions in response to cognate P-Ag ligands and different cytokine stimulation^51^. Moreover, recent studies in mice highlight that γδ TCR triggering of innate-like lymphocytes induced a state of TCR hyporesponsiveness in which T-cell activation could be induced by TCR-extrinsic stimuli^6^ Unfortunately, a systematic comparison of the TCR responsiveness of naive vs. effector Vγ9^−^ Vδ2^+^ T cells within one tissue was not possible, due to the strong dominance of naive Vγ9^−^ Vδ2^+^ T cells in peripheral blood and effector Vγ9^−^ Vδ2^+^ T cells in liver. However, importantly within the Vδ1^+^ T-cell compartment, CD27^lo/neg^ effectors display a far quicker response to TCR stimulation than CD27^hi^ naive cells^11^. This observation emphasises the strong distinction between innate-like and adaptive subsets within the γδ T-cell compartment.

Human Vδ2^+^ T cells therefore comprise two separate subsets, which reflect distinct paradigms in human γδ T-cell biology and also have distinct roles in antimicrobial immunity. Vγ9^+^ Vδ2^+^ T cells appear to represent an innate-like subset, most likely originating via gestational selection, which is ‘pre-armed’ from birth with a semi-invariant TCR repertoire that includes a high level of public Vγ TCR chains, permitting polyclonal TCR-mediated responses to microbial P-Ag, as well as to innate cytokines independently of the TCR. However, the Vγ9^+^ Vδ2^+^ TCR repertoire diversity may provide the potential for clonotype-specific responses potentially targeted at specific microbial antigenic challenges. In contrast, Vγ9^−^ Vδ2^+^ T cells, which are P-Ag-unreactive, represent a previously ill-defined unconventional T-cell compartment that exhibits many of the key hallmarks of an adaptive immunobiology, including clonal expansion and differentiation to effector lymphocytes from an initially naive-like and TCR diverse T-cell pool, including in response to acute viral infection. In doing so, Vγ9^−^ Vδ2^+^ T cells most likely contribute to both peripheral blood and tissue immunosurveillance.

## Methods

### Ethical approval and samples

Peripheral blood samples were obtained from healthy donors who had provided written informed consent for sample collection and subsequent analysis; project approval for this aspect of the study was granted by the NRES Committee West Midlands ethical board (REC reference 14/WM/1254). Samples from patients undergoing renal transplantation were obtained at the Academic Medical Centre, Amsterdam; the medical ethics committee of the Academic Medical Center, Amsterdam, approved this arm of the study and all subjects provided written informed consent in accordance with the Declaration of Helsinki. Longitudinal heparinized blood samples were collected from CMV-seronegative patients who developed primary CMV infection after receiving a renal transplant from a CMV-seropositive donor. As controls, longitudinal PBMC from age matched CMV-seronegative patients who remained CMV-seronegative during the first 5 years following renal transplantation were used. All patients were EBV-seropositive. The course of CMV infection was followed by longitudinal 2-weekly PCR for CMV viral load, and seroconversion was confirmed by detection of CMV-specific IgM and IgG. The patients did not receive CMV prophylaxis, but once the CMV load reached 10^-4^ copies/ml, the MMF dose was halved and Valcyte was given. Only patient 1014 experienced CMV-related symptoms: CMV gastritis. All but one patient were treated with a basic immunosuppressive regimen comprising of CD25 mAb (basiliximab) induction treatment, prednisolone, cyclosporine A and MMF. Patient 261, however, was treated with one dose of prednisolone upon transplantation with a kidney from an identical twin, whereafter all immunosuppression was ceased. Liver infiltrating T cells were isolated from explanted diseased human liver tissues from patients undergoing liver transplantation for inflammatory liver diseases, including primary biliary cholangits, primary sclerosing cholangitis, alcoholic liver disease, and autoimmune hepatitis (Local Research Ethics Committee reference no. 98/CA5192) or normal liver samples from donor liver tissue surplus to clinical requirements (Local Research Ethics Committee reference no. 06/Q2708/11). Umbilical cord blood units were obtained from the Anthony Nolan Cell Therapy Centre Nottingham (ANCTC) under generic tissue bank ethics held by ANCTC and extended to the researchers under a material transfer agreement (MTA).

### T-cell isolation, culture and activation

PBMC were isolated from heparinised venous blood by lymphoprep© (Stem Cell Technologies) density gradient centrifugation as per the manufacturers instructions. For proliferation of T cells, PBMC were labelled with 0.3 µM carboxyfluorescein succinimidyl ester (CFSE) (eBioscience) and cultured with 10 nM HMB-PP (Sigma) or CD3/CD28 T activator beads (Invitrogen) for 7 days in RPMI-1640 medium (Invitrogen) supplemented with 2 mM L-glutamine, 1% sodium pyruvate, 50 μg/ml penicillin/streptomycin (Invitrogen) and 10% fetal calf serum (Sigma).

### Antibodies and flow cytometry

For total and single-cell sorting of Vδ2^+^ populations, PBMC were labelled with anti-CD3 (UCHT1; Biolegend; 1:100), TCR αβ (BW242/412; Miltenyi; 1:200), TCR Vδ2 (123R3; Miltenyi; 1:200), CD27 (M-T271; 1:200), CD45RA (HI100; 1:200); both Biolegend, and populations were sorted on an ARIA III Fusion (BD) (Supplementary Fig. 4a). For repertoire analysis, Vδ2^+^ T-cell populations were sorted directly into RNAlater (Sigma). For phenotypic analysis, freshly isolated or frozen PBMC, or cultured cells were labelled with Zombie Aqua viability dye (Biolegend), and then subsequently stained (Supplementary Fig. 4b) for cell surface antigens with antibodies directed against CD3 (UCHT1 or HIT3a; 1:100), CD8 (SK1; 1:200), CD45RA (HI100; 1:200), CD27 (M-T271; 1:200), CCR7 (G043H7; 1:50), IL7Rα (A019D5; 1:100), CD28 (28.2; 1:100), CX_3_CR1 (2A9-1; 1:100), CD16 (3G8; 1:150), CD69 (FN50; 1:100), TCR Vδ2 (B6; 1:100), TCR αβ (IP26; 1:50); all Biolegend. TCR Vγ9 (IMMU360; 1:400); Beckman Coulter. TCR Vδ1 (REA173; 1:200) and TCR Vδ2 (123R3; 1:200); Miltenyi, or αGalCer loaded CD1d dextramers (ProImmune; 1:20). For intracellular staining, after surface antibody staining cells were fixed in IC Fixation buffer (eBioscience) and stained in Permeabilisation Buffer (eBioscience) with antibodies directed against Granzyme A (CBO9; 1:100) and Granzyme B (GB11; 1:100); Biolegend). Cells were acquired on an LSR Fortessa X20 (BD) and data analysed with FlowJo V10.2 (TreeStar).

### TCR repertoire analysis

RNA was purified from sorted cells (adult Vδ2^+^: 25,000 cells; cord blood Vδ2^+^: 2,400 - 8,800 cells) using an RNAmicro plus kit (Qiagen) according to the manufacturer’s instructions. For high throughput deep sequencing of γδ TCRs, we used amplicon rescued multiplex (ARM)-PCR and a MiSeq (Illumina) next-generation sequencer (NGS)^52^ to analyse all sorted Vδ2^+^ T-cell populations. As detailed in patent WO2009137255A2, a modified version of a protocol devised by Han et al.^53^ was used, involving initial first-round RT-PCR using high concentrations of gene-specific primers, followed by use of universal primers for the exponential phase of amplification, allowing deep, quantitative and non-biased amplification of TCR γ and TCR δ sequences. All cDNA synthesis, amplification, NGS library preparation and sequencing were performed by iRepertoire, Inc. (Huntsville, USA). We analysed positively sorted αβ TCR^-^ Vδ2^+^ γδ T cells from 7 healthy donors and 4 umbilical cord blood units (Anthony Nolan Trust, Nottingham).

### Single-cell TCR sequencing

PBMC were labelled as above and Vδ2^+^ T cells were single cell sorted directly into individual wells in a 96-well plate containing 2 µl of Superscript VILO cDNA synthesis kit reaction mix (ThermoFisher) containing 0.1% Triton X-100, and incubated according to manufacturer’s instructions. TCRγ and TCRδ cDNAs were amplified by two rounds of nested PCR using GoTaq mastermix (Promega) and primers for Vδ2, TCTGGGCAGGAGTCATGT (external) and GAAAGGAGAAGCGATCGGTAAC (internal); for Cδ GCAGGATCAAACTCTGTTATCTTC (external) and TCCTTCACCAGACAAGCGAC (internal); for Vγ1–8 CTGGTACCTACACCAGGAGGGGAAGG (external) and TGTGTTGGAATCAGGAVTCAG (internal); for Vγ9 AGAGAGACCTGGTGAAGTCATACA (external) and GGTGGATAGGATACCTGAAACG (internal) and for Cγ CTGACGATACATCTGTGTTCTTTG (external) and AATCGTGTTGCTCTTCTTTTCTT (internal). PCR products were separated on 1.2% agarose gels, and products of successful reactions were incubated with ExoSAP-IT PCR cleanup enzyme (Affymetrix) before sequencing with BigDye Terminator v3.1 (Applied Biosystems) following manufacturer’s instructions and running on an ABI 3730 capillary sequencer (Functional Genomics Facility, University of Birmingham).

### TCR repertoire data analysis

V, D and J gene usage and CDR3 sequences were identified and assigned and tree maps generated using iRweb tools (iRepertoire, Inc, Huntsville, AL, USA)^54^. Tree maps show each unique CDR3 as a coloured rectangle, the size of each rectangle corresponds to each CDR3s abundance within the repertoire and the positioning is determined by the V region usage. To determine the 10 most prevalent shared CDR3γ9 sequences, the first 10 most dominant sequences by frequency were filtered from each donors CDR3γ9 protein lists. These sequences were then ordered by frequency for sequences that were shared between >2 donors (to create a hierarchy of 10 common sequences) and uncommon highly amplified sequences were included in the final analysis. For more detailed analysis of the TCR repertoire, datasets were processed using the MiXCR software package^55^ to further correct for PCR and sequencing errors. Diversity metrics, clonotype overlap and gene usage were plotted in R, by VDJTools^56^.

### TCR sequence analyses

The CDR3 sequence was defined as the amino acids between the second Cysteine of the V region and the conserved Phenylalanine of the J region, according to IMGT. CDR3 sequences shown in tables include the conserved Cysteine and Phenylalanine, but only the amino acids between these residues are counted for CDR3 length analysis and for analysis of residues at position 5. N and P nucleotides were identified using the IMGT Junction Analysis tool^57,58^. Neonatal Vδ2 sequence logos were generated on the Seq2Logo server^59^ in Shannon format without the use of pseudo counts, and give a visual representation of amino acids enriched at different positions in the observed CDR3δ2 sequences. The different amino acids are coloured according to physicochemical properties (acidic (DE), red; basic (RKH), blue; hydrophobic (ACFILMPVW), black; and neutral (NSGTY), green). For TCRδ2 sequences, the ten most abundant clonotypes of 14–16 amino acids using either Jδ3 or Jδ1 from each donor were aligned using Clustal Omega52 with default parameters, before logo generation. Narrower bars in the sequence logo correspond to gaps in the sequences.

### Statistical analysis

Each data set was assessed for normality using Shapiro–Wilk normality test. Differences between columns were analysed by two-tailed Student’s *t*-tests for normally distributed data and Mann–Whitney for non-parametric data. Differences between groups were analysed using one-way ANOVA with Dunnett’s or Tukey’s post tests for normally distributed data or with Kruskal–Wallis with Tukey’s post tests for non-parametric data and RM two-way ANOVA with Tukey’s post tests was used when comparing groups with independent variables. *P < 0.05, **P < 0.01, ***P < 0.001 and ****P < 0.0001. Correlation was assessed for non-parametric data by Spearman correlation. Tabulated data were analysed in Graphpad PRISM 7 (Graphpad Software, Inc.).

### Data availability

All data are available from the authors upon request. The sequence data that support the findings of this study have been deposited in the NIH NCBI sequence read archive (SRA) database with the primary accession code SRP113556 and SRP096009, for Vδ2^+^ and Vδ1^+^ TCR repertoires respectively.

## Electronic supplementary material

Supplementary Information
