# Supplementary material for: The human Vδ2+ T-cell compartment comprises distinct innate-like Vγ9+ and adaptive Vγ9- subsets
Source: Nat Commun. 2018 May 2;9:1760. doi: 10.1038/s41467-018-04076-0 (PMC5932074; doi:10.1038/s41467-018-04076-0)
Supplement: Supplementary file 1 — Supplementary Information [file 41467_2018_4076_MOESM1_ESM.pdf]

The human Vδ2$^+$ T cell compartment comprises distinct innate-like Vγ9$^+$ and adaptive Vγ9$^-$ subsets

Martin S. Davey, Carrie R. Willcox, Stuart Hunter, Sofya A. Kasatskaya, Ester B. Remmerswaal, Mahboob Salim, Fiyaz Mohammed, Frederike J. Bemelman, Dmitriy M. Chudakov, Ye H. Oo and Benjamin E. Willcox

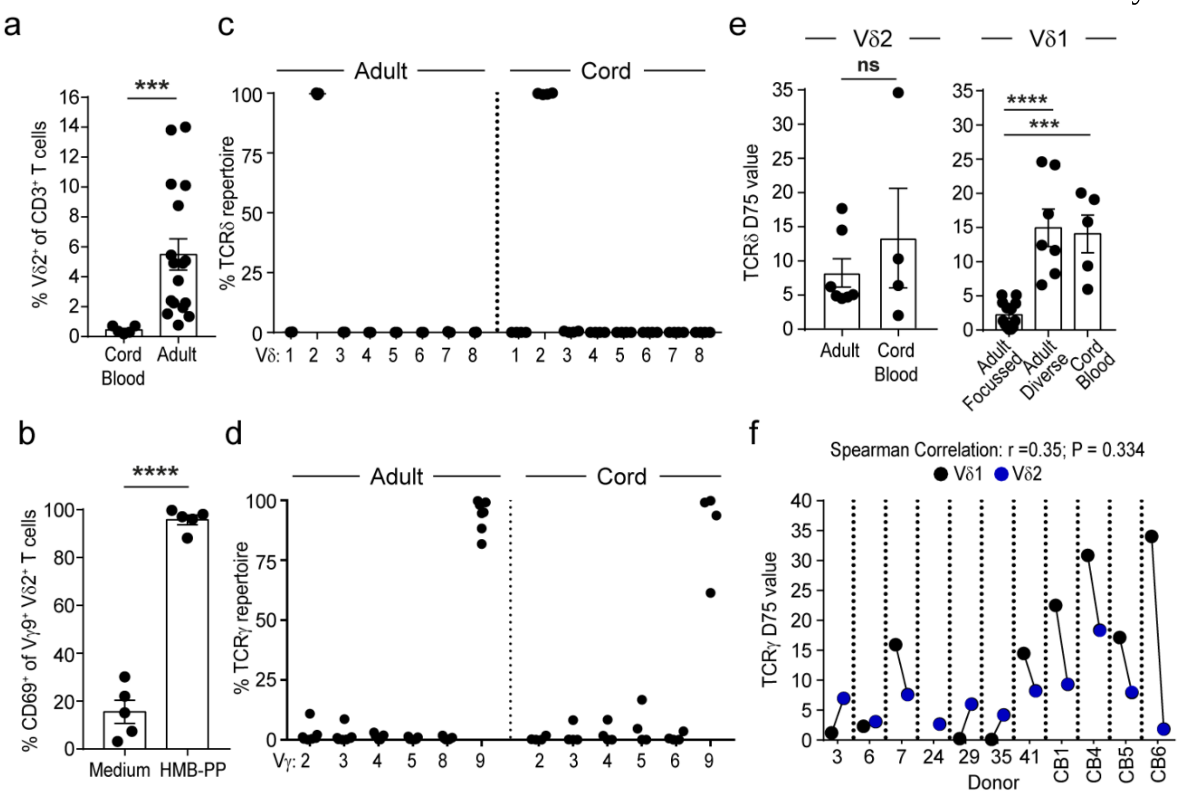

Supplementary Figure 1. Profiling of Vδ2$^+$ T cells and TCR repertoires in adults and neonates. (**a**) Frequency of Vδ2$^+$ cells in CD3$^+$ T cells from cord blood samples (n=5) and adult peripheral blood (n=18). (**b**) Frequency of CD69$^+$ Vγ9$^+$ Vδ2$^+$ T cells from PBMC cultured in the presence of medium or 10 nM HMB-PP for 18 hours (n=5). (**c**) Vδ, (**d**) Vγ usage in Vδ2$^+$ TCR repertoires from adult peripheral blood (n=7) and cord blood samples (n=4). (**e**) Comparison of TCRδ D75 metrics from Vδ2$^+$ T cells from adult peripheral blood, cord blood samples (adult: n=7; cord blood: n=4) and Vδ1$^+$ TCR repertoires (adult focussed: n=13; adult diverse: n=7; cord blood: n=5). (**f**) Comparison of TCRγ D75 metrics from paired Vδ2$^+$ and Vδ1$^+$ TCR repertoires from the same adult peripheral blood and cord blood samples. Adult donor 24 had no matched Vδ1$^+$ TCR repertoire data. Error bars indicate means ± SEM; * $P < 0.05$; *** $P < 0.001$; **** $P < 0.0001$; p-values were determined by Mann-Whitney t-test (a and e); Student's t-test (b) and One-way ANOVA with Tukey's post hoc testing (e).

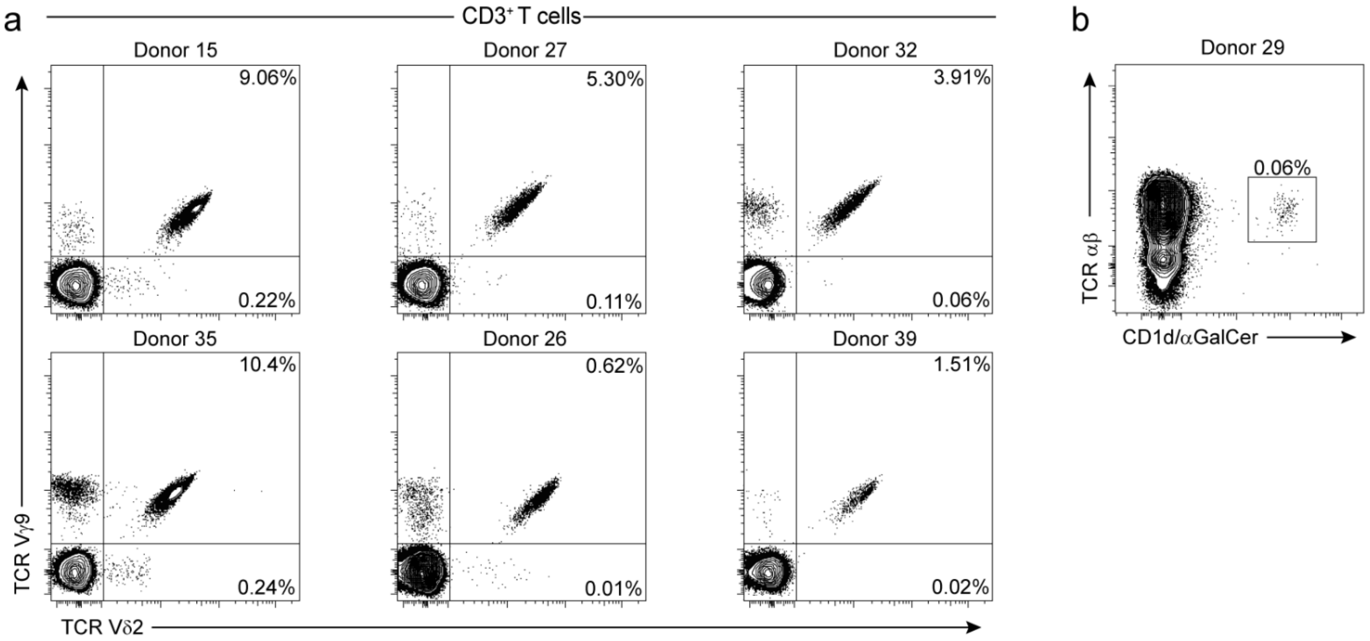

Supplementary Figure 2. Identification of Vγ9$^-$ Vδ2$^+$ T cells and NKT cells in healthy adults. (**a**) Representative identification of Vγ9$^-$ Vδ2$^+$ T cells by monoclonal antibodies directed against TCR Vγ9 and TCR Vδ2 in CD3$^+$ T cells from adult peripheral blood samples (n=18). (**b**) Representative identification of NKT cells with an αGalCer loaded CD1d-dextramer in CD3$^+$ T cells from adult peripheral blood samples (n=5).

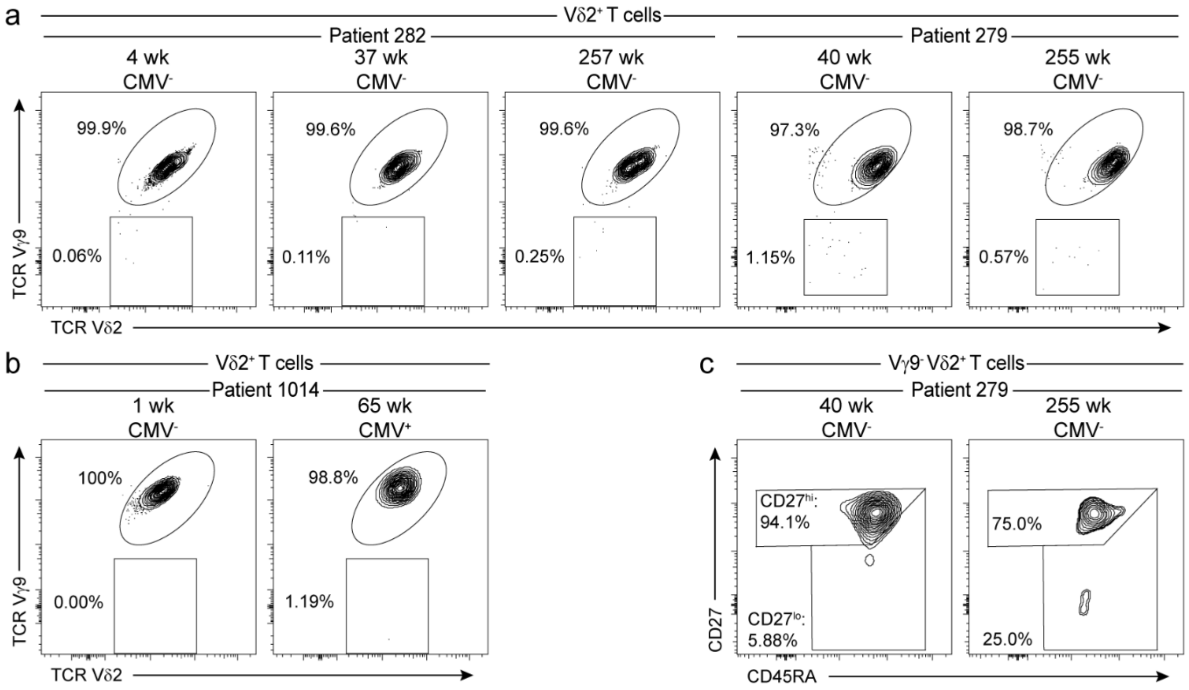

Supplementary Figure 3. Vγ9- Vδ2+ T cells in kidney transplant patients. (**a**) Identification of Vγ9- cells within total Vδ2+ T cells in two CMV-seronegative kidney transplant patients who did not develop post-operative acute-CMV infection and (**b**) a patient who was infected with CMV and seroconverted at 5 weeks post-transplantation. (**c**) Analysis of CD27 and CD45RA T cell memory marker expression on detectable Vγ9- Vδ2+ T cells populations from CMV-seronegative patient 279 at 40 and 255 weeks post-kidney transplant.

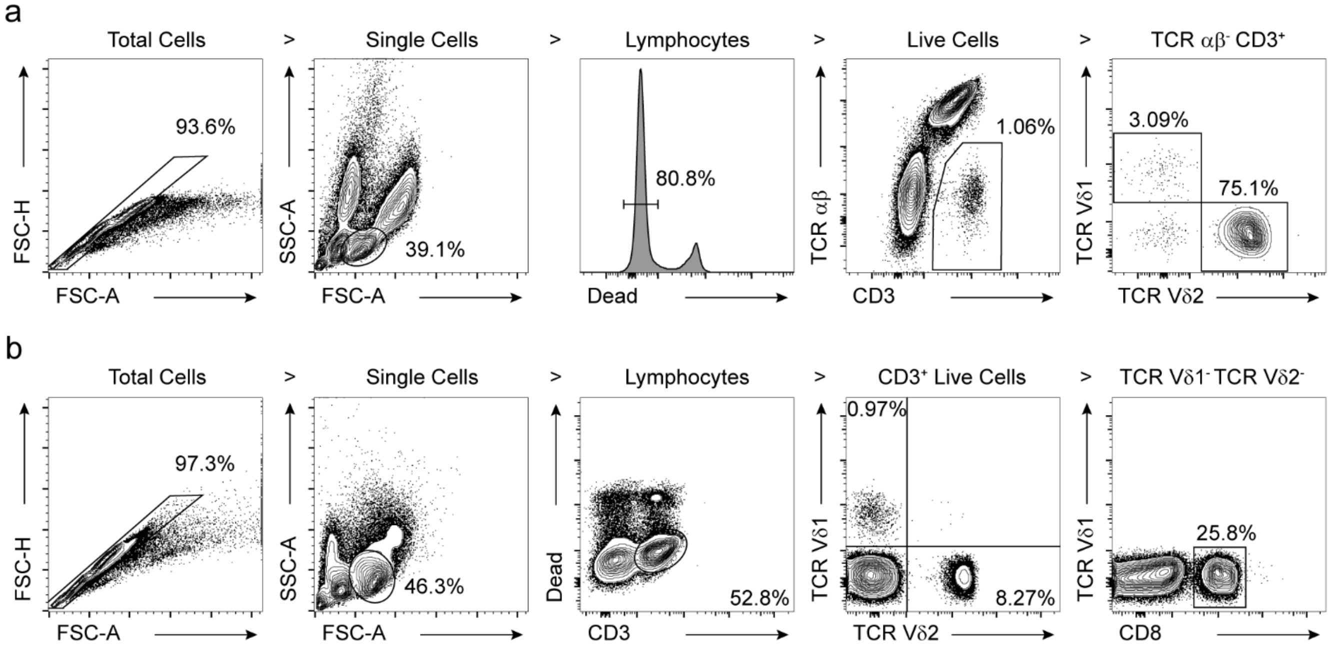

Supplementary Figure 4. Gating strategy for identification and sorting of T cell populations. (**a**) Representative flow cytometry plots show the gating strategy used to identify single cells>lymphocytes>live cells>CD3$^+$ TCR αβ$^-$ T cells>Vδ2$^+$ populations for cell sorting (related to Figure 1, 2 and Supplementary Figure 1). (**b**) Representative identification of single cells>lymphocytes>live cells CD3$^+$>Vδ1$^+$, Vδ2$^+$ and Vδ1$^-$ Vδ2$^-$ CD8$^+$ T cells from peripheral blood and liver tissue samples (related to Figure 2, 3, 4, 5, 6, Supplementary Figure 2 and 3).

Cord Blood 1	%	Cord Blood 4	%	Cord Blood 5	%	Cord Blood 6	%
CACDWGSSWDTRQMFF	6.2	CACDWGSSWDTRQMFF	8.2	CACDILGDTDKLIF	5.4	CACDSLGDTSPDKLIF	11.6
CACDILGDTDKLIF	3.1	CACDTGGYSWDTRQMFF	3.4	CACDKLGDTDKLIF	4.1	CACDRGIRRSWDTRQMFF	10.4
CACDVLGDTDKLIF	1.5	CACDILGDTDKLIF	1.9	CACDTVLGDTWDTRQMFF	3.0	CACDGKSTSSWDTRQMFF	9.7
CACDVLGDTAQLFF	1.5	CACDTVLGDTWDTRQMFF	1.4	CACDWGSSWDTRQMFF	2.5	CACDTVYWGIRSSWDTRQMFF	9.7
CACDRGYYTDKLIF	1.2	CACDVLGDTDKLIF	1.3	CACDVLGDRHDKLIF	2.2	CACDSLGDTWDTRQMFF	9.7
CACDVLGDLTAQLFF	1.1	CACDYWGSSWDTRQMFF	1.1	CACDVLGDTDKLIF	2.1	CACDTGGRWGIRLWDTRQMFF	9.1
CACDGILTAQLFF	0.9	CACDTWGSSWDTRQMFF	0.9	CACDNTGGSWDTRQMFF	1.8	CACDTATPLESGGYEVGTDKLIF	7.5
CACDTWGYTDKLIF	0.9	CACDILGDTWDTRQMFF	0.6	CACDTVIGGIRPYTDKLIF	1.7	CACDTGKWDTRQMFF	7.3
CACDSGIWTAQLFF	0.9	CACDWGTWDTRQMFF	0.6	CACDTGGYWDTRQMFF	1.6	CACDTGDTLWDTRQMFF	3.8
CACDILGDTWDTRQMFF	0.8	CACDTWGTDKLIF	0.6	CACDTLGVLDKLIF	1.5	CACDTGWSSWDTRQMFF	3.3

Supplementary Table 1. Top 10 most prevalent TCRδ sequences in cord blood Vδ2$^+$ T cells. The table depicts CDR3δ2 amino acid sequence and frequency occupied in the total TCRδ repertoire from four cord blood donors.

CDR3δ2	CDR3 length	TRDD	TRDJ	P nt	N nt	CB01	CB04	CB05	CB06
CACDWGSSWDTRQMFF	14	3	3	1	0	>5%	>5%	2.5-5%	ND
CACDTAGGYSWDTRQMFF	16	3	3	1	0	ND	<1%	ND	1-2.5%
CACDTWDTRQMFF	11	--	3	0	0	ND	<1%	1-2.5%	ND
CACDWGTWDTRQMFF	13	3	3	0	0	ND	<1%	ND	1-2.5%
CACDTGDTLWDTRQMFF	15	3	3	1	1	ND	<1%	<1%	2.5-5%
CACDTVLGDTWDTRQMFF	16	3	3	2	0	<1%	1-2.5%	2.5-5%	ND
CACDNTGGSWDTRQMFF	15	3	3	1	1	<1%	<1%	1-2.5%	ND
CACDTGGYWDTRQMFF	14	3	3	0	0	ND	<1%	1-2.5%	ND
CACDTWGSSWDTRQMFF	15	3	3	1	0	ND	<1%	<1%	ND
CACDYWGSSWDTRQMFF	15	3	3	2	0	<1%	1-2.5%	1-2.5%	ND
CACDTGGYSWDTRQMFF	15	3	3	0	0	<1%	2.5-5%	<1%	ND
CACDTAGGSWDTRQMFF	15	3	3	1	0	ND	<1%	<1%	ND
CACDTAGSSWDTRQMFF	15	3	3	2	1	ND	<1%	<1%	ND
CACDTVGGSSWDTRQMFF	16	3	3	3	2	<1%	<1%	<1%	ND
CACDTYWGSSWDTRQMFF	16	3	3	2	0	<1%	<1%	1-2.5%	ND
CACDTGGYSSWDTRQMFF	16	3	3	2	0	<1%	<1%	<1%	ND
CACDILGDTDKLIF	12	3	1	1	0	2.5-5%	1-2.5%	>5%	ND
CACDVLGDTDKLIF	12	3	1	2	0	1-2.5%	1-2.5%	1-2.5%	ND
CACDTLGDTDKLIF	12	3	1	0	0	<1%	<1%	1-2.5%	ND

Frequency (shading)	
black	**>5%**
dark grey	**2.5-5%**
mid grey	**1-2.5%**
light grey	**<1%**
white	**ND**

Supplementary Table 2. Shared TCRδ sequences in cord blood Vδ2^{+} T cells. The table depicts public CDR3δ2 sequences, amino acid length, Dδ, and Jδ gene segments used, N-nucleotide, P-nucleotide addition and relative frequency within each TCRδ repertoire. Sequences were analysed using IMGT Junction Analysis and are from four cord blood donors. ND = not detected.
